# Prevalence of severe *Plasmodium knowlesi* infection and risk factors related to severe complications compared with non-severe *P. knowlesi* and severe *P. falciparum* malaria: a systematic review and meta-analysis

**DOI:** 10.1186/s40249-020-00727-x

**Published:** 2020-07-29

**Authors:** Manas Kotepui, Kwuntida Uthaisar Kotepui, Giovanni D. Milanez, Frederick R. Masangkay

**Affiliations:** 1 Medical Technology, School of Allied Health Sciences, Walailak University, Tha Sala, Nakhon Si Thammarat, Thailand; 2 Department of Medical Technology, Far Eastern University, Manila, Philippines

**Keywords:** *Plasmodium knowlesi*, Severe malaria, *Plasmodium falciparum*, Prevalence

## Abstract

**Background:**

*Plasmodium knowlesi* is a potential cause of severe and fatal malaria, but comprehensive studies of its pooled prevalence and risk factors are lacking. This study aimed to explore the prevalence and risk factors related to severe *P. knowlesi* infection.

**Methods:**

A systematic review was conducted by retrieving all published articles on severe *P. knowlesi* available in Web of Science (ISI), Scopus, and PubMed (MEDLINE). Titles, abstracts, and full-text articles were screened, and any irrelevant studies were excluded. The random-effects model was used to compute the pooled prevalence estimate of severe *P. knowlesi* infection by a metaprop command provided in STATA software. Differences in demographic characteristics, clinical characteristics, and laboratory data were analysed using Review Manager Version 5.3 software for patients in the following groups: 1) patients with severe and non-severe *P. knowlesi* infection and 2) patients with severe *P. knowlesi* and severe *P. falciparum* infection.

**Results:**

Out of the 2382 studies retrieved from the three databases, seven studies with a total enrolment of 1124 patients with *P. knowlesi* infections were eligible to be included in this systematic review and meta-analysis. The pooled prevalence estimate of severe *P. knowlesi* infection was 19% (95% *CI:* 11–27%, *I*^2^ = 93.7%). Severe acute kidney injuries (AKI) (77 cases, 45.6%), jaundice (71 cases, 42%), and hyperparasitaemia (55 cases, 32.5%) were the common clinical manifestations found among patients with severe complications. In comparison to non-severe *P. knowlesi* infections, patients with severe *P. knowlesi* infections had significantly higher age, leucocyte count, and parasitaemia levels (*P* < 0.05). In comparison to patients with severe *P. falciparum* infections, patients with severe *P. knowlesi* infections had significantly higher age, neutrophil count, and creatinine levels (*P* < 0.05).

**Conclusions:**

This systematic review and meta-analysis demonstrated a high proportion of severe *P. knowlesi* infections. Patients with severe *P. knowlesi* infections had higher age, leucocyte count, and parasitaemia levels than those with non-severe *P. knowlesi* infections. In addition, patients with severe *P. knowlesi* infections had higher age, neutrophil count, and creatinine levels than those with severe *P. falciparum* infections.

## Background

*Plasmodium knowlesi* is a naturally occurring malarial parasite in monkeys [1] and is acknowledged as the fifth human malarial parasite, especially among travellers returning from Southeast Asian countries or among people living in endemic areas where monkeys are present, such as in Malaysian Borneo [2]. The natural reservoir hosts of *P. knowlesi* are the long-tailed macaque (*Macaca fascicularis*) and the pig-tailed macaque (*Macaca nemestrina*, *Macaca leonine*) [3–6]. The geographical distribution of *P. knowlesi* is found only in Southeast Asian countries because its natural hosts and the female *Anopheles* mosquito vectors are very limited and specifically exist in these areas [4]. A previous study demonstrated that the mosquito vectors in the *Leucosphyrus* Complex transmitted *P. knowlesi* in southern Asia, while the *Dirus* Complex transmitted *P. knowlesi* in Vietnam, Myanmar, Laos, Cambodia and Thailand [6, 7]. At present, there is no evidence indicating that *P. knowlesi* is transmitted from human to human but rather through mosquito bites [8]. Infections of *P. knowlesi* occur through quotidian (24-hour schizogonic cycles) malarial parasites and lead to severe and lethal manifestations.

The first case of *P. knowlesi* infection in humans was reported by Singh et al. in Sarawak, Malaysia, in patients previously misdiagnosed with *P. malariae* infections [2]. The morphology of *P. knowlesi* is similar to that of *P. malariae,* a quartan malarial parasite causing non-severe human malaria [9]. This similarity contributes to the problem that technicians encounter when identifying species of malaria under light microscopy. The evidence was supported by a study by William et al., which demonstrated that some infections with *P. knowlesi* confirmed by polymerase chain reaction (PCR) were miss-detected as *P. malariae* by microscopic methods (76%) [10]. The rapid diagnostic tests (RDTs) were commercially available for the detection of *P. knowlesi,* but they were insensitive in cases of low parasitaemia and were unable to distinguish *P. knowlesi* from *P. vivax* [11, 12]. Hence, confirmation of *P. knowlesi* requires a molecular technique, such as the nested PCR assay. A previous study demonstrated that *P. knowlesi* misdiagnosis as *P. malariae* caused severe malaria and resulted in death because of inappropriate treatment [13]. It is very important to emphasize to physicians the need to recognize non-falciparum malaria as a potential cause of severe and fatal malaria in addition to *P. vivax* and to prevent the fatal outcome of patients by administering intravenous therapy with artesunate or quinine as the first drug of choice for severe malaria, especially caused by *P. knowlesi*. Although *P. knowlesi* is a potential cause of severe and fatal malaria, comprehensive studies of its pooled prevalence and risk factors are lacking. In addition, a meta-analysis determining the difference in the prevalence estimate of severe *P. knowlesi* infection among studies conducted on *P. knowlesi* is needed. Therefore, this study aimed to estimate the prevalence and risk factors related to severe *P. knowlesi* malaria. In addition, the comparison of demographic characteristics, clinical data, and laboratory data between severe *P. knowlesi* and severe *P. falciparum* infection was evaluated to predict disease severity in co-endemic primary care settings of *P. knowlesi*.

## Methods

### Article searching strategy

This systematic review and meta-analysis was performed according to the Preferred Reporting Items for Systematic Review and Meta-Analysis (PRISMA) guidelines (PRISMA 2009 Checklist S1). Web of Science (ISI), Scopus, and PubMed were queried for studies before March 16, 2019, using the terms “(severe OR complicated OR complication) AND ((“*Plasmodium knowlesi*”) OR knowlesi)” (Table S1). This search was limited to human studies in the English language. The population, intervention, control, and outcomes (PICO) format was used to define the selection criteria: P, patients with severe *P. knowlesi*; I, severe malaria; C, patients with severe *P. falciparum*; and O, demographic characteristics, clinical characteristics, and laboratory profiles. The present study included cross-sectional studies that reported on the prevalence of *P. knowlesi* malaria in humans. Studies were excluded if they were case reports or case series, animal studies, clinical drug studies, co-infection studies, commentaries, experimental studies, reviews, or reports with incomplete data. Additionally, any study using the same data reported by groups of authors was excluded from the present study.

### Data extraction

Two reviewers (MK and KUK) selected and extracted the data from the included studies independently. We used the Endnote software X7 (Clarivate Analytics, Philadelphia, USA) to manage the references. First, we removed any duplicate papers retrieved from the three databases. Then, we screened the titles and abstracts according to the inclusion criteria. Finally, we assessed all full-text articles for detailed assessments for further data extraction. The data extraction was conducted using the Microsoft Excel 2010 (Microsoft Corporation, Washington, USA) and included data on the primary author, year of publication, study area, study design, demographic characteristics of patients (age and sex), and clinical characteristics (duration of fever, signs and symptoms, temperature in degrees Celsius [°C], blood pressure, pulse rate, respiratory rate, and previous malaria exposure). Laboratory profiles including parasite count, haemoglobin, leucocyte count, platelet count, creatinine, sodium, bilirubin, glucose, albumin, aspartate aminotransferase (AST), and alanine aminotransferase (ALT) were additionally collected. The malaria endemicity of the years when the included studies were conducted by their authors was demonstrated by the slide positivity rate as reported by the World Malaria Report 2010–2014 [14–18]. Disagreements in data extraction were resolved through discussion or by consulting a third reviewer (FRM).

### Criteria of severe *P. knowlesi* malaria

The criteria of severe *P. knowlesi* malaria were defined by the World Health Organization (WHO) [19].

### Characteristics of the included studies

A quality assessment was performed following the quality assessment tool developed by the Newcastle-Ottawa Scale (NOS) for assessing the quality of nonrandomized studies in meta-analysis [20]. It provided a star system with a maximum of nine stars to judge the included studies on three broad perspectives: the selection of the study groups, the comparability of the groups, and the ascertainment of outcome among the studies.

### Data synthesis

The primary outcome of the present study was the pooled prevalence of severe *P. knowlesi* infection. The data analysis for the pooled prevalence began with entry of the extracted data into the Excel sheet and to the STATA Statistical Software version 15.0 (StataCorp LLC, Texas, USA). The number of patients with severe *P. knowlesi* and total cases of *P. knowlesi* were imported and analysed using a command “metaprop case population, random/fixed” provided in the STATA software. A metaprop command allowed Freeman-Tukey double arcsine transformation to stabilize the variances. The random-effects or fixed-effect model could be used to compute the pooled estimate, depending on the heterogeneity among the included studies [21]. The results are presented with the pooled prevalence along with the 95% confidence interval (*CI*).

The secondary outcome of the present study was to identify factors associated with severe *P. knowlesi* malaria using the demographic characteristics, clinical characteristics, and laboratory profiles of patients with severe and non-severe *P. knowlesi* malaria. The third outcome of the present study was to compare the demographic characteristics, clinical characteristics, and laboratory profiles between patients with severe *P. knowlesi* and severe *P. falciparum* malaria. The results of the second and third outcomes were presented as the pooled mean difference (MD) with 95% *CI* for continuous outcomes and odds ratio along with 95% *CI* for categorical outcomes. The data heterogeneity was assessed using Cochrane Q (chi-square) and Moran’s *I*^2^ (inconsistency). The *I*^2^ was quantified as low, moderate, and high, with upper limits of 25, 50, and 75% for *I*^2^, respectively. If moderate or high heterogeneity was presented by Moran’s *I*^2^, the random-effects model was used to calculate the pooled estimates. If a low heterogeneity (*I*^2^ < 25%) was presented, the fixed-effect model was used to calculate the pooled estimates. The subgroup analysis of study sites was performed to demonstrate whether the transmission intensity between Sarawak and Sabah, the area with the highest transmission intensity of all malaria cases and the area with the highest cases of *P. knowlesi* in Malaysia during 2013–2017, affected the pooled prevalence estimate or affected the risk of severe *P. knowlesi* infection in the meta-analysis. The analysis of the second and third outcomes was performed using the Review Manager 5.3 (The Cochrane Collaboration, London, UK). The meta-regression analysis of a covariate, namely, malaria endemicity (slide positive rate), between severe *P. knowlesi* and non-severe *P. knowlesi* was performed using STATA Statistical Software version 15.0.

### Publication bias

The funnel plot was used to demonstrate any symmetrical funnel plot among patients with severe *P. knowlesi* infection. The evaluation of publication bias among the included studies helped explore the underlying cause of data heterogeneity if it was present in the included studies.

## Results

### Search results and study characteristics

**Fig. 1:**
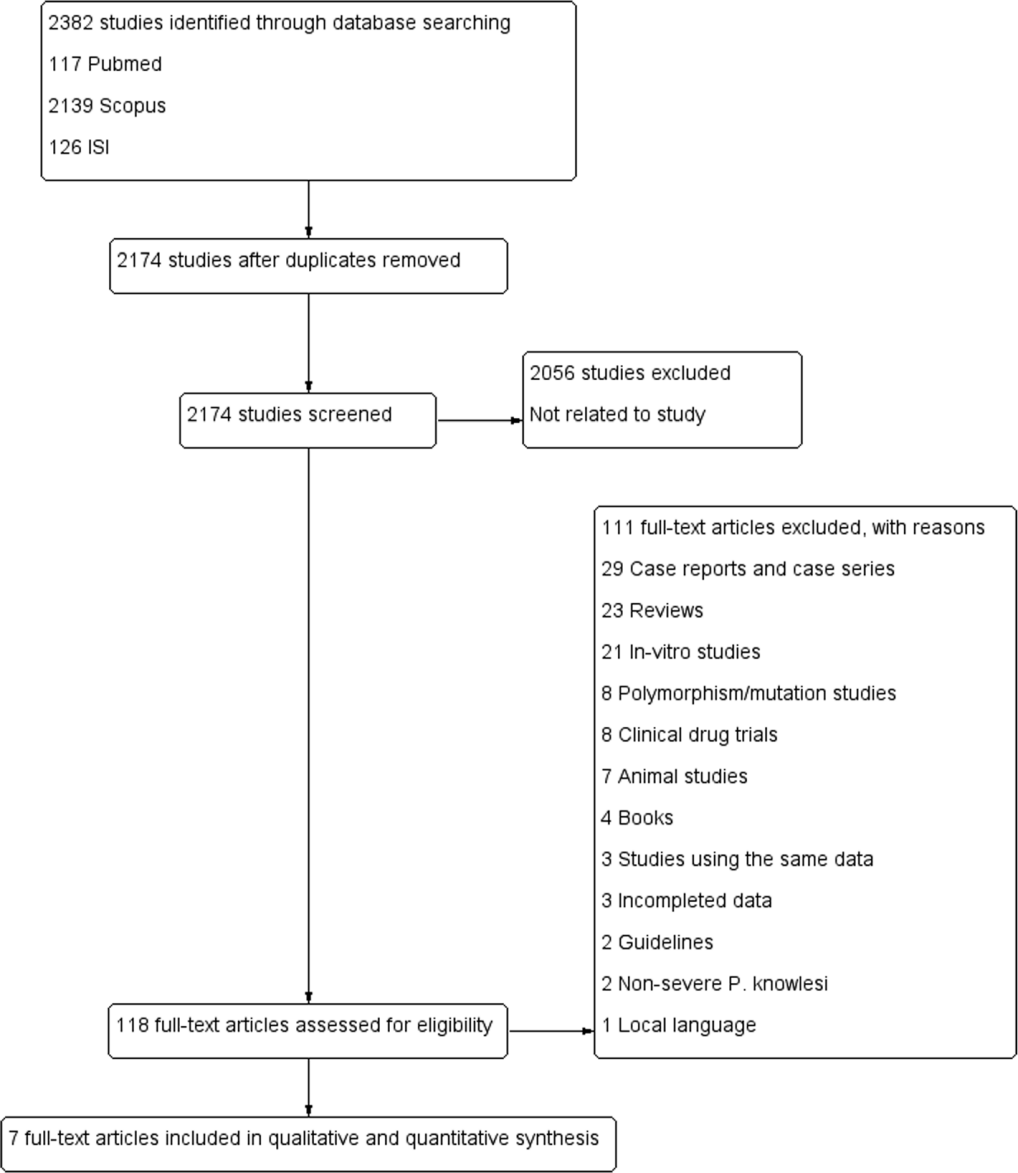
PRISMA diagram. Flow chart for study selection

A total of 2382 studies were retrieved from PubMed (*n* = 117), Scopus (*n* = 2139), and Web of Science (*n* = 126) databases (Fig. 1), of which 208 studies were found to be duplicates. Of the 2174 articles reviewed, 2056 articles were excluded because they were not related to *P. knowlesi*. Of the 118 full-text articles reviewed, 111 articles were excluded for various reasons, as shown in Fig. 1. Seven studies were included for systematic review and meta-analysis.

The characteristics of the seven studies included in this study are presented in Table 1, Table S2, and Table S3. Table 1 presents demographic profiles and the number of patients with *P. knowlesi* and *P. falciparum* infections. Of those seven studies, all cases were conducted in Malaysia during 2006–2013. Four studies [22, 26, 28, 29] were conducted in Sabah, whereas the other three studies were conducted in Sarawak [23, 24, 27], Malaysian Borneo. The seven studies included prospective observational studies [22, 28, 29], cross-sectional observational studies [23, 24], a retrospective study [26], and a case control study [27]. All seven studies reported a total of 1124 patients with *P. knowlesi* infections. Of those 1124 patients, 169 patients (15%) had severe complications. Most of the severe complications found in patients included severe acute kidney injury (AKI) (77 cases, 45.6%), jaundice (71 cases, 42%), and hyperparasitaemia (55 cases, 32.5%). The number of patients with *P. falciparum* infections included a total of 394 patients in five studies [22–24, 28, 29]. Of those 394 patients, 44 patients (11.2%) had severe complications. Common clinical manifestations found in patients included jaundice (24 cases, 54.5%), shock (15 cases, 34.1%), and respiratory distress (11 cases, 25%). Other complications of severe *P. knowlesi* and *P. falciparum* infections are shown in Table 1.

**Table 1:** Characteristics of the included studies

No.	Author, publication year, reference number	Study area (years of the survey)	Study design	Malaria endemicity	Number of ***P. falciparum*** infections	Severe ***P. falciparum*** infection (%)	Number of ***P. knowlesi*** infections	Severe ***P. knowlesi*** (%)	Complications of ***P. falciparum***	Complications of ***P. knowlesi***
1.	Barber et al., 2013, [11]	Sabah, Malaysia (2010–2011)	A prospective observational study	Slide positive rate 2010 (0.4%), 2011 (0.3%) (World Malaria Report 2012) Average: 0.35%	122	13	130	38	Hyperparasitaemia (2) Jaundice (9) Respiratory distress (4) Severe AKI (3) Shock (6) Metabolic acidosis (4) Severe anaemia (2) Abnormal bleeding (1)	Hyperparasitaemia (18) Jaundice (20) Respiratory distress (14) Severe AKI (9) Shock (13) Metabolic acidosis (4) Severe anaemia (2) Abnormal bleeding (2)
2.	Barber et al., 2017, [22]	Sabah, Malaysia (2010–2012)	A prospective observational study	Slide positive rate 2010 (0.4%), 2011 (0.3%) (World Malaria Report 2012), 2012 (0.3%) (World Malaria Report 2014) Average: 0.33%	130	21	146	47	Hyperparasitaemia (3) Jaundice (12) Respiratory distress (4) Severe AKI (3) Shock (7) Metabolic acidosis (4) Severe anaemia (2) Abnormal bleeding (1) Hypoglycaemia (1)	Hyperparasitaemia (24) Jaundice (21) Respiratory distress (14) Severe AKI (11) Shock (11) Metabolic acidosis (4) Severe anaemia (5) Abnormal bleeding (5) One severity criterion (19) Two severity criterion (17) Three or more criterion (11)
3.	Cox-Singh et al., 2011, [23]	Kapit Division, Sawarak, Malaysia (2006–2009)	A cross-sectional observationa study	Slide positive rate 2006 (0.38%), 2007 (0.35%), 2008 (0.47%), 2009 (0.45%) (World Malaria Report 2010) Average: 0.41%	22	5	94	9	Hyperparasitaemia (2) Jaundice (3) Respiratory distress (3) Severe AKI (3) Shock (2) Hypoglycaemia (1)	Hyperparasitaemia (2) Jaundice (4) Respiratory distress (2) Severe anaemia (2)
4.	Daneshvar et al., 2009, [24]	Kapit Division, Sawarak, Malaysia (2006–2008)	A cross-sectional observationalstudy	Slide positive rate 2006 (0.38%), 2007 (0.35%), 2008 (0.47%) (World Malaria Report 2010) Average: 0.4%	24	NA	107	8	NA	Hyperparasitaemia (3) Jaundice (3) Respiratory distress [4] Severe AKI (3) Shock (2) Hypoglycaemia (1)
5.	Grigg et al., 2017, [25]	Sabah, Malaysia (2011–2013)	A prospective observational study	Slide positive rate 2011 (0.3%) (World Malaria Report 2012), 2012 (0.3%), 2013 (0.2%) (World Malaria Report 2014) Average: 0.27%	96	5	481	28	NA	Hyperparasitaemia (8) Jaundice (8) Respiratory distress (2) Severe AKI (10) Shock (5) Metabolic acidosis (3) Severe anaemia (8) Abnormal bleeding (1) Two or more criterion (12)
6.	William et al., 2011, [26]	Sabah, Malaysia (2007–2009)	A retrospective study	Slide positive rate 2007 (0.35%), 2008 (0.47%), 2009 (0.45%) (World Malaria Report 2010) Average: 0.42%	NA	NA	56	22	NA	Jaundice (9) Respiratory distress (13) Severe AKI (12) Shock (12)
7.	Willmann et al., 2012, [27]	Sarikei and Sibu, Sawarak, Malaysia (2007–2010)	A case control study	Slide positive rate 2007 (0.35%), 2008 (0.47%), 2009 (0.45%) (World Malaria Report 2010), 2010 (0.4%) (World Malaria Report 2012) Average: 0.42%	NA	NA	110	17	NA	Jaundice (6) Respiratory distress (3) Severe AKI (32) Shock (1) Severe anaemia (1)
	Total				394	44	1124	169	Jaundice (24, 54.5%) Shock (15, 34.1%) Respiratory distress (11, 25%) Severe AKI (9, 20.5%) Metabolic acidosis (8, 18.2%) Hyperparasitaemia (7, 15.9%) Severe anaemia (4, 9.1%) Abnormal bleeding (2, 4.54%) Hypoglycaemia (2, 4.54%)	Severe AKI (77, 45.6%) Jaundice (71, 42%) Hyperparasitaemia (55, 32.5%) Respiratory distress (52, 30.8) Shock (45, 26.6%) Metabolic acidosis (11, 6.5%) Severe anaemia (18, 10.7%) Abnormal bleeding (8, 4.73%)

*AKI* Acute kidney injuries, *NA* Not assessed

### Prevalence of severe *P. knowlesi* malaria

**Fig. 2:**
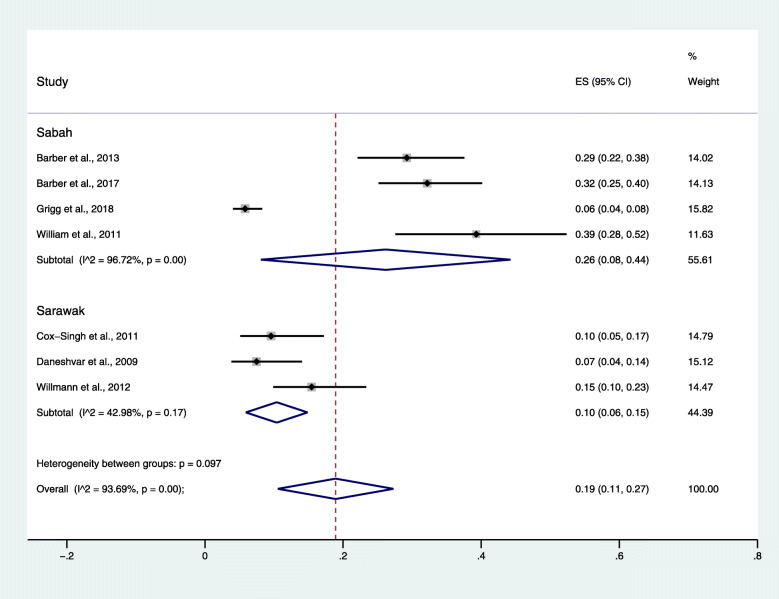
The pooled prevalence of severe *Plasmodium knowlesi* malaria

The pooled prevalence of severe *P. knowlesi* malaria was 19% (95% *CI*: 11–27, *I*^2^ = 93.7%), calculated by a random-effects model since high heterogeneity (Moran’s *I*^2^: 93.7%) was found (Fig. 2). The highest prevalence of severe *P. knowlesi* malaria was reported in studies by William et al. (39, 95% *CI*: 28–52%) [26] and by Barber et al. (32, 95% *CI*: 25–40, *I*^2^ = 93.7%) [22], while the lowest prevalence of severe *P. knowlesi* malaria was reported in a study by Grigg et al. (6, 95% *CI*: 4–8%) [29]. The subgroup analysis of study sites demonstrated that the pooled prevalence estimate of the included studies conducted in Sabah (26, 95% *CI*: 8–44%, *I*^2^ = 96.7%) was higher than that of the studies conducted in Sarawak (10, 95% *CI*: 6–15%, *I*^2^ = 43%).

### Risk factors associated with severe *P. knowlesi* malaria

**Fig. 3:**
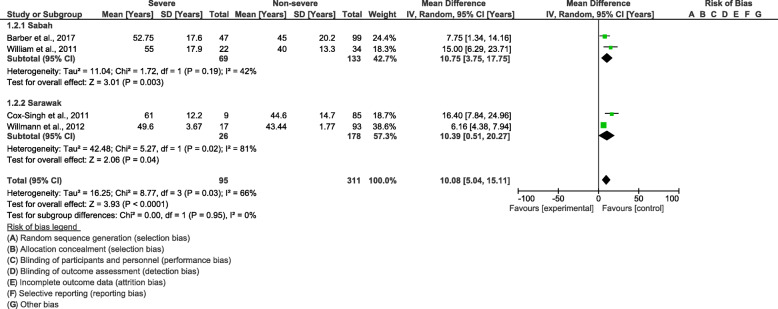
The pooled mean difference of age between patients with severe and non-severe *Plasmodium knowlesi* infections

Cochrane Q and Moran’s *I*^2^ analysis of age (*I*^2^ = 66%), leucocyte count (*I*^2^ = 54%), and parasitaemia level (*I*^2^ = 91%) between patients with severe and non-severe *P. knowlesi* infections demonstrated that medium and high heterogeneities were found among the included studies; therefore, the random-effects model was used to calculate the pooled MD along with 95% *CI*. In comparison to patients with non-severe *P. knowlesi* infections, the results demonstrated that patients with severe *P. knowlesi* infections were significantly older than patients with non-severe *P. knowlesi* infections (*P <* 0.0001, MD: 10.08, 95% *CI*: 5.04–15.11, *I*^2^ = 66%) (Fig. 3). The subgroup analysis of study sites demonstrated that a significantly older age was found in both the included studies conducted in Sabah (*P =* 0.03, MD: 10.75, 95% *CI*: 3.75–17.75, *I*^2^ = 42%) and those studies conducted in Sarawak (*P =* 0.04, MD: 10.08, 95% *CI*: 0.51–20.27, *I*^2^ = 81). The meta-regression analysis using the slide positive rate as a covariate revealed that the slide positive rate was not a confounding factor for the age difference (*P =* 0.718, *t* = 0.42, 95% *CI*: − 10.8–13.1).

**Fig. 4:**
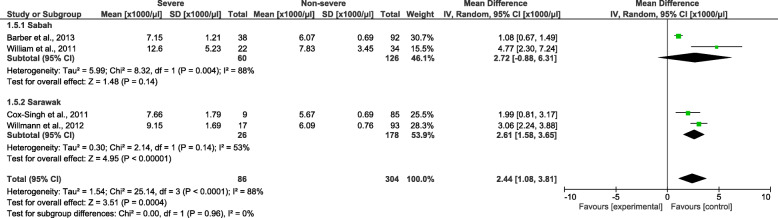
The pooled mean difference of leucocyte count between patients with severe and non-severe *Plasmodium knowlesi* infections

Patients with severe *P. knowlesi* infections had a significantly higher leucocyte count than patients with non-severe *P. knowlesi* infections (*P =* 0.0004, MD: 2440, 95% *CI*: 1080–3810, *I*^2^ = 88%) (Fig. 4). The subgroup analysis of study sites demonstrated that a significantly higher leucocyte count was found in the included studies conducted in Sarawak (*P <* 0.00001, MD: 2610, 95% *CI*: 1580–3650, *I*^2^ = 53%) but not in the included studies conducted in Sabah (*P* = 0.14, MD: 2720, 95% *CI*: − 880–6310, *I*^2^ = 88%). The meta-regression analysis using the slide positive rate as a covariate revealed that the slide positive rate was not a confounding factor for the leucocyte count difference (*P =* 0.516, *t* = 0.78, 95% *CI*: −63–91).

**Fig. 5:**
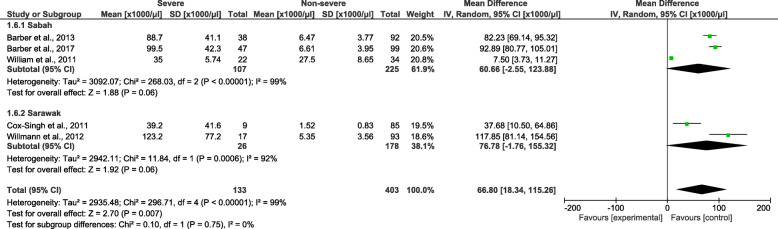
The pooled mean difference of parasitaemia level between patients with severe and non-severe *Plasmodium knowlesi* infections

Patients with severe *P. knowlesi* infections had a significantly higher parasitaemia level than patients with non-severe *P. knowlesi* infections (*P* = 0.007, MD: 66 800, 95% *CI*: 18 340–115 260) (Fig. 5). The subgroup analysis of study sites demonstrated that no subgroup difference was observed (*P =* 0.75), and the subgroup analysis demonstrated that no significantly higher parasitaemia level was found in the included studies conducted in Sarawak (*P =* 0.06, MD: 76780, 95% *CI*: − 1760–155 320, *I*^2^ = 92%) compared to the included studies conducted in Sabah (*P =* 0.06, MD: 60 660, 95% *CI*: − 2550–123 880, *I*^2^ = 99%). The meta-regression analysis using the slide positive rate as a covariate revealed that the slide positive rate was not a confounding factor for the parasitaemia level difference (*P =* 0.375, *t* = − 1.04, 95% *CI*: − 60.7–30.8).

**Fig. 6:**
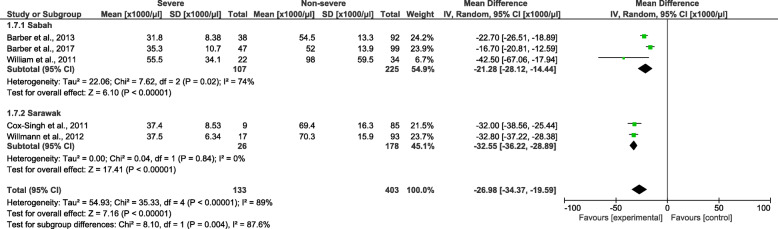
The pooled mean difference of platelet count between patients with severe and non-severe *Plasmodium knowlesi* infections

Cochrane Q and Moran’s *I*^2^ analysis of platelet counts between patients with severe and non-severe *P. knowlesi* infections demonstrated a high level of heterogeneity (*I*^2^ = 89%); therefore, the random-effects model was used to calculate the pooled MD along with 95% *CI*. In comparison to patients with non-severe *P. knowlesi* infections, patients with severe *P. knowlesi* infections had a significantly lower platelet count (*P* < 0.00001, MD: − 26 980, 95% *CI*: − 34 370–−19 590) (Fig. 6). The subgroup analysis of study sites demonstrated that a subgroup difference was observed (*P* = 0.004), and a significantly lower platelet count was found in both the included studies conducted in Sabah (*P* < 0.00001, MD: − 21 280, 95% *CI*: − 28 120–14 440, *I*^2^ = 74%) and those studies conducted in Sarawak (*P* < 0.00001, MD: − 32 550, 95% *CI*: − 36 220–28 890, *I*^2^ = 0%). The meta-regression analysis using the slide positive rate as a covariate revealed that the slide positive rate was not a confounding factor for the platelet count difference (*P* = 0.848, *t* = − 0.21, 95% *CI*: −10.2–22).

### Severe *P. knowlesi* and severe *P. falciparum* malaria

**Fig. 7:**
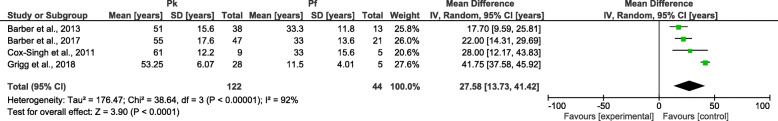
The pooled mean difference of age between patients with severe *Plasmodium knowlesi* and severe *P. falciparum* infections

**Fig. 8:**
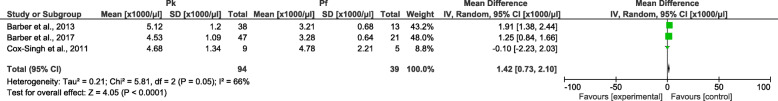
The pooled mean difference of neutrophil counts between patients with severe *Plasmodium knowlesi* and severe *P. falciparum* infections

**Fig. 9:**
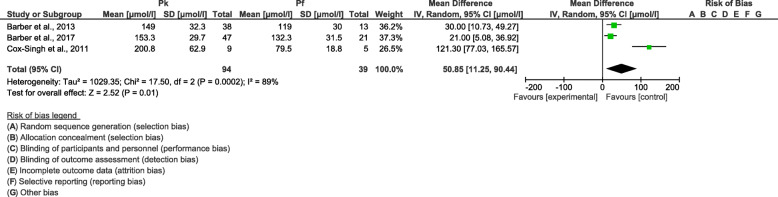
The pooled mean difference of creatinine levels between patients with severe *Plasmodium knowlesi* and severe *P. falciparum* infections

Cochrane Q and Moran’s *I*^2^ analysis of age (*I*^2^ = 92%), neutrophil count (*I*^2^ = 66%), and creatinine level (*I*^2^ = 89%) between patients with severe *P. knowlesi* and severe *P. falciparum* infections demonstrated that medium and high heterogeneities were found among the included studies; therefore, the random-effects model was used to calculate the pooled MD along with 95% *CI*. In comparison to patients with severe *P. falciparum* infections, patients with severe *P. knowlesi* infections were older (*P* < 0.0001, MD: 27.6, 95% *CI*: 13.7–41.4) (Fig. 7), had a higher neutrophil count (*P* < 0.0001, MD: 1420, 95% *CI*: 730–2100) (Fig. 8), and had a higher creatinine level (*P* = 0.01, MD: 50.9, 95% *CI*: 11.3–90.4) (Fig. 9). The meta-regression analysis using the slide positive rate as a covariate revealed that the slide positive rate was not a confounding factor for age (*P* = 0.297, *t* = − 1.40, 95% *CI*: − 139.8–71.3), neutrophil count (*P* = 0.363, *t* = − 1.56, 95% *CI*: − 169.9–132.7), or creatinine level (*P* = 0.289, *t* = 2.05, 95% *CI*: − 100.8–139.6).

### Occupational risk of patients with *P. knowlesi* infections

The proportion of farmers infected with *P. knowlesi* was not different from those infected with *P. falciparum* (*P* = 0.19, *OR* = 5.54, 95% *CI*: 0.43–71.7) (Supplementary Figure 1). The proportion of plantation workers infected with *P. knowlesi* was not different from those infected with *P. falciparum* (*P =* 0.33, *OR* = 0.19, 95% *CI*: 0.01–5.33) (Supplementary Figure 2).

### Quality of included studies and publication bias

All seven studies included in the present study had max scores of nine stars according to the NOS guidelines, indicating good quality. The funnel plot analysis among the included studies could not be performed as it required a minimum of 10 studies for the analysis [30].

## Discussion

This study provided prevalence data and risk factors related to severe *P. knowlesi* malaria in comparison with severe *P. falciparum* malaria. All seven included studies conducted in Malaysia reported on severe *P. knowlesi* complications. The subgroup analysis of study sites demonstrated that the pooled prevalence estimate of the included studies conducted in Sabah (26%) was higher than that of the studies conducted in Sarawak (10%). Previous studies reported that *P. knowlesi*, not *P. falciparum* or *P. vivax,* was the most common cause of malaria in Malaysia, particularly in Sabah and Sarawak [31, 32]. Another study revealed that the highest prevalence of *P. knowlesi* malaria was observed in wild macaques (87%) and long-tailed macaques (50%) living in Sarawak, Malaysian Borneo [4, 33]. The most recent retrospective study, which reviewed the malaria-confirmed cases of the Ministry of Health, Malaysia between January 2013 and December 2017, revealed that the highest average incidence rate (AIR) was found in Sarawak (0.420 per 1000 population) and Sabah (0.383 per 1000 population) from 2013 to 2017 [34]. Our results suggested that although the prevalence of *P. knowlesi* was higher in Sarawak, the highest proportion of severe malaria caused by *P. knowlesi* was found in another state, Sabah. The result of our study was supported by the average mortality rate per 100 000 patients with *P. knowlesi* being higher in Sarawak (0.074) than in Sabah (0.064) [34].

In addition to being endemic in Malaysia, the presence of *P. knowlesi* in wild long-tailed macaques (19%) was also reported from the island of Palawan, the Philippines [35]. In humans, asymptomatic infections with *P. knowlesi* parasites were also found in individuals living close to forested areas in Cambodia (0.59%) [36]. A previous study suggested that the microscopic appearance of *P. malariae* should be reported as *P. knowlesi* to increase recognition and be treated with intravenous artesunate for severe *P. knowlesi* malaria [37]. In Malaysia, current *P. knowlesi* malaria management guidelines recommend an initial treatment with intravenous artesunate for any patients > 50 years of age or with a parasitaemia level > 20 000/μl [28, 37].

The most common complications of severe *P. knowlesi* malaria found in the present study were severe AKI, jaundice, and hyperparasitaemia. The results were similar to the largest study of *P. knowlesi* malaria conducted in three hospitals in Malaysia, where AKI was frequently observed [29]. The mechanisms regarding the pathology and physiology of severe malaria from *P. knowlesi* in humans are not well understood. However, even a low parasite density from *P. knowlesi* infections can lead to severe diseases [16]. In severe *P. knowlesi* malaria, hyperparasitaemia has been defined as a parasite count of 100000 parasites/μl or higher [28, 38]. The life cycle of *P. knowlesi* in the human host is a 24-hour life cycle in which it rapidly reproduces at the asexual stage at a high parasitaemia level more than *P. falciparum* [28]. For AKI, the mechanism in severe malaria from *P. knowlesi* is still unknown; however, microvascular sequestration similar to that in *P. falciparum* infections might be the cause that plays a role in kidney injury [39].

The meta-analysis demonstrated that older patients had more severe complications from *P. knowlesi* infections than younger patients, which was related to the low risk of anaemia and high risk of thrombocytopenia [2, 28, 29]. However, a previous study suggested that *P. knowlesi* infected a wide range of ages from young teenagers to mature adults, which was in accordance with a study in Sarawak, Malaysia [24]. Two previous studies demonstrated that the mean age of patients was 45 years [24, 28]. A possible reason that might explain why *P. knowlesi* infections occurred more frequently among older patients was the strong correlation of increasing age with increasing parasitaemia [28]. Increasing age was associated with increasing systemic inflammation and impairment of microvascular function, contributing to severe malaria in older adults with *P. knowlesi* infections [22]. This mechanism-related severe malaria in older patients compared to younger patients might be similar to that in *P. falciparum* malaria, where endothelial activation increases with age [40]. In addition, the limited outdoor activities of children may be related to the low evidence of *P. knowlesi* infections among these age groups [32]. A previous study also suggested that the transmission of *P. knowlesi* over a wide age range might be related to the presence of family clusters [41]. The results of the present study demonstrated that the proportion of occupations (farmers and plantation workers) was similar in both *P. knowlesi* and *P. falciparum* infections. This demonstrated that patients who worked as farmers and plantation workers could be infected with either *P. knowlesi* or *P. falciparum* at the same proportion. However, a previous study indicated that *P. knowlesi* parasitaemia was significantly higher in farmers and plantation workers than in other occupations [22]. Moreover, farming was a risk factor for the infection of *P. knowlesi* malaria but not for non-*P. knowlesi* malaria [25]. Previous studies demonstrated that the risk of *P. knowlesi* infection was related to human behaviours, such as higher forest exposure or working in agricultural areas [25, 28]. A previous study also showed that males demonstrated a four-fold greater risk of symptomatic *P knowlesi* infection and that the risk of infection by *P. knowlesi* and non-*P. knowlesi* was comparable in males [25]. In contrast, sex was not significantly associated with *P. knowlesi* seropositivity in a study by Fornace et al., 2018, who conducted a study in northern Sabah, Malaysia, and Palawan, the Philippines [42]. The most recent study demonstrated that patients with *P. knowlesi* infection were work-related, including Armed Forces and security personnel, boatmen and researchers, Department of Forestry officers, border patrol officers, and non-work-related, including peri-domestic transmission [43].

Comparing patients with severe and non-severe *P. knowlesi* malaria, the present study demonstrated that patients with severe *P. knowlesi* malaria had a higher leucocyte count and parasitaemia level than those with non-severe *P. knowlesi* malaria. The subgroup analysis of study sites demonstrated that a significantly higher leucocyte count was found in the included studies conducted in Sawarak but not in the included studies conducted in Sabah. These results demonstrated that *P. knowlesi* patients in Sawarak had an immune response that was strongly correlated to *P. knowlesi* infection, as the leucocyte differential count and neutrophil counts were strongly correlated with *P. knowlesi* parasitaemia [23]. The results from a previous study reported that more than 80% of patients with *P. knowlesi* malaria had a parasitaemia level greater than 100 000 parasites/μl [28]. They also indicated that neutrophil counts were correlated with *P. knowlesi* parasitaemia levels, suggesting that severe *P. knowlesi* malaria can lead to parasite-induced inflammation such as lung injury [28]. The present study found a lower platelet count in patients with severe *P. knowlesi* malaria than in those with non-severe *P. knowlesi* malaria. A previous study reported thrombocytopenia (84.9%) among patients with *P. falciparum* and *P. vivax* infections [44]. The thrombocytopenia in the present study might be related to a high parasitaemia level, as presented by a study in Thailand [45]. A previous study reported that patients with *P. knowlesi* malaria demonstrated a decrease in coagulation system markers [46] or an increase in the proportion of platelet binding to infected red cells [47]. The risk factors for death from *P. knowlesi* infection demonstrated in Sabah, Malaysia, were female sex, age ≥ 45 years, cardiovascular disease, microscopic misdiagnosis, and delay in administrating intravenous treatment [48].

The results of the present study demonstrated that severe complications in patients with *P. knowlesi* malaria were higher than those with *P. falciparum* malaria. This result supported the conclusion of a previous study in Sabah, Malaysia, which demonstrated severe *P. knowlesi* malaria at 29% with a greater than 3-fold risk of severity when compared to *P. falciparum* malaria [28]. Comparing patients with severe *P. knowlesi* and severe *P. falciparum* malaria, patients with severe *P. knowlesi* infections were older than patients with severe *P. falciparum* malaria. This result was similar to a previous study demonstrating that patients with *P. knowlesi* malaria were older than those with *P. falciparum* or *P. vivax* malaria [28]. Previous studies have suggested that severe *P. knowlesi* infections and death have not been reported in children, unlike severe *P. falciparum* infections [31].

The present study demonstrated that patients with severe *P. knowlesi* malaria had a higher neutrophil count than patients with severe *P. falciparum* malaria. These results were supported by a previous study that revealed that neutrophil counts strongly correlated with *P. knowlesi* parasitaemia [23]. They suggested that the strong association between *P. knowlesi* parasitaemia and neutrophils involved the cytokines IL-1ra and IL-10, which were related to anti-inflammatory effects on the patient’s response to infection [23]. A previous study demonstrated a high neutrophil count with a high neutrophil/lymphocyte (NL) ratio, demonstrating that neutrophil and lymphocyte counts were the most important leucocyte changes during malaria infections [45]. A high neutrophil count among patients with *P. knowlesi* malaria might be due to a consistent correlation between high neutrophil counts and high parasite densities in *P. falciparum-*infected patients when compared to those with low and moderate parasitaemia groups [45, 49]. Previous studies also demonstrated that the median neutrophil count on admission was higher in severe *P. knowlesi* than in non-severe *P. knowlesi* infections [23, 27]. The present study demonstrated that patients with severe *P. knowlesi* malaria had a higher creatinine level than those with severe *P. falciparum* malaria. These results demonstrated the association between a higher creatinine level and a higher proportion of severe AKI among patients with severe *P. knowlesi* malaria. As the risk of severity occurred in a high proportion compared to *P. falciparum* malaria in Malaysia, all patients recognized with severe malaria due to *P. knowlesi* were given intravenous artesunate as a first-line treatment upon admission to reduce the potential of mortality [28].

The present study had limitations. First, only a few articles on severe *P. knowlesi* infections could be selected and extracted, so the pooled analysis needs to be carefully interpreted. Second, some clinical and laboratory data were missing among the seven included studies, causing a low statistical power to infer the differences between severe *P. knowlesi* and severe *P. falciparum* malaria. Third, subgroup analysis of study sites between Sabah and Sarawak in the meta-analysis of severe *P. knowlesi* and severe *P. falciparum* infections could not be performed, as three studies included in the analysis were conducted in Sabah and only one included study was conducted in Sarawak. Fourth, some confounding factors might have affected the haematological analysis of the present study, such as demographic factors, malaria immunity, underlying diseases, and the haemoglobinopathy of patients with malaria. Due to the limitations of the data on haemoglobinopathies and G6PD deficiency of the study populations reported in the included studies, the effect of haemoglobinopathies and G6PD deficiency on the severity or laboratory parameters between patients with severe *P. knowlesi* and non-severe *P. knowlesi* infections could not be examined in the present study.

## Conclusions

This systematic review demonstrated a high rate of severe *P. knowlesi* infections. Patients with severe *P. knowlesi* malaria were older, had a higher leucocyte count, and had a lower platelet count than those with non-severe *P. knowlesi* malaria. Patients with severe *P. knowlesi* infections were older, had a higher neutrophil count, and had a higher creatinine level than those with severe *P. falciparum* infections. Future work must aim to explore the risk factors with a higher number of enrolled studies to aid in the understanding of severe *P. knowlesi* infections in malaria-endemic settings.

## Supplementary information

**Additional file 1.** PRISMA 2009 Checklist S1.

**Additional file 2: Table S1.** Search term.

**Additional file 3: Table S2.** Characteristic of severe and non-severe complications of *Plasmodium knowlesi.*

**Additional file 4: Table S3.** Characteristic of severe *Plasmodium knowlesi* and *P. falciparum.*

**Additional file 5: Figure S1.** The proportion of farmers infected with *Plasmodium knowlesi* and *P. falciparum.*

**Additional file 6: Figure S2.** The proportion of plantation workers infected with *Plasmodium knowlesi* and *P. falciparum.*

## Data Availability

All data related to the present study are available in the manuscript and supplementary files.

## Abbreviations

AKI: Acute kidney injuries
ALT: Alanine aminotransferase
AST: Aspartate aminotransferase
*CI*: Confidence interval
MD: Mean difference
NOS: Newcastle-Ottawa Scale
PCR: Polymerase chain reaction
PICO: Population, intervention, control, and outcomes
PRISMA: Preferred Reporting Items for Systematic Review and Meta-Analysis
RDTs: Rapid diagnostic tests
WHO: World Health Organization
