# Supplementary material for: Prevalence of severe Plasmodium knowlesi infection and risk factors related to severe complications compared with non-severe P. knowlesi and severe P. falciparum malaria: a systematic review and meta-analysis
Source: Infect Dis Poverty. 2020 Jul 29;9:106. doi: 10.1186/s40249-020-00727-x (PMC7392650; doi:10.1186/s40249-020-00727-x)
Supplement: Supplementary file 2 — Additional file 2: Table S1. Search term. [file 40249_2020_727_MOESM2_ESM.docx]

**Prevalence and factors related to severe *Plasmodium knowlesi* infection: A systematic review and analysis**

**Search term**

(severe[All Fields] OR complicated[All Fields] OR complication[All Fields]) AND ("Plasmodium knowlesi"[All Fields] OR knowlesi[All Fields])
