# Supplementary material for: Prevalence of severe Plasmodium knowlesi infection and risk factors related to severe complications compared with non-severe P. knowlesi and severe P. falciparum malaria: a systematic review and meta-analysis
Source: Infect Dis Poverty. 2020 Jul 29;9:106. doi: 10.1186/s40249-020-00727-x (PMC7392650; doi:10.1186/s40249-020-00727-x)
Supplement: Supplementary file 3 — Additional file 3: Table S2. Characteristic of severe and non-severe complications of Plasmodium knowlesi. [file 40249_2020_727_MOESM3_ESM.docx]

**Table S2** Characteristic of severe and non-severe complications of *P.knowlesi*

| No. | Author |  | Age, mean (range/IQR) | Male sex, No. (%) | Fever duration | Systolic blood pressure, mm Hg, mean (SD) | Pulse rate, beats/min, mean (SD) | Respiratory rate, breaths/min, mean (SD) | Temperature, °C, mean (SD) | Previous malaria (self-reported) | White blood cell counts (10^3^/μl) | Parasite count (10^3^parasites/μl) | Platelet counts (10^3^ platelets/μl) |
| --- | --- | --- | --- | --- | --- | --- | --- | --- | --- | --- | --- | --- | --- |
| 1. | Barber et al., 2013 | Severe (38) | Median 55 (20–74) | 30 (78.9) |  |  |  |  |  |  | 6.60 (5.60–9.80) | 80.3 (25.9–  168.3) | 29 (20–49) |
|  |  | Non-severe (92) |  |  |  |  |  |  |  |  | 6.05 (4.95–7.25) | 4.84 (1.58–  14.6 | 51 (35–81) |
| 2. | Barber et al., 2017 | Severe (47) | Median 55 (20–81) | 35 (75) | 6 (3–7) | 112 (16) | 90 (16) | 28 (6) | 37.5 (1.1) | 15 (32) |  | 104  (21.7–168.3) | 32 (20–57) |
|  |  | Non-severe (99) | Median 42 (13–83) | 73 (74) | 5 (3–7) | 120 (20) | 89 (15) | 26 (6) | 37.6 (1.1) | 26 (26) |  | 4.84  (1.56–15.2) | 50 (30–78) |
| 3. | Cox-Singh et al., 2011 | Severe (9) | 61:12.2 (36 - 73) | 33% | 5(3.25 - 6.75) | 74(11.69) |  | 28(21 - 32.5) | 37.4(37 - 38.4) |  | 7.4(4.85 – 11) | 2.17 (4.3 – 148.3) | 35 (25- 54.5) |
|  |  | Non-severe (85) | 44.6: 14.7 (16 - 79) | 57% | 4(3 - 7) | 89(9.43) |  | 27(23 - 31) | 37.6(36.85 - 38.5) |  | 5.6 (4.6 – 6.9) | 1.03 (0.34 – 3.14) | 65 (45.5 - 102) |
| 4. | Daneshvar et al., 2009 | Severe (8) | 58.4 (36-73) | 3 (33) |  |  |  |  |  |  |  |  |  |
| 5. | Grigg et al., 2018 | Severe (28) | Median 53 (43–64) | 19 (68) |  |  |  |  |  | 36% |  | 42.23 (17.2–103.6) |  |
|  |  | Non-severe (453) |  | 23% |  |  |  |  |  | 19% |  |  |  |
| 6. | William et al., 2011 | Severe (22) | 57 (22–84) | 14 (73.7) | 5.2 (2–7) | 74 (42–106) | 100 (76–130) | 26 (15–50) | 37.6(36.7–39.2) | 0 | 12.7 (3.5–21.6) | 40 (20–40) | 40 (12–130) |
|  |  | Non-severe (34) | 37 (20–66) | 31 (89.2) | 5.0 (1–30) | 85 (61–106) | 95 (69–151) | 20 (14–26) | 38.2(36.5–41) | 1 | 6.3 (3.4–15.3) | 30 (10–40) | 72 (21–227) |
| 7. | Willmann et al., 2012 | Severe (17) | 49.59 (43.24 – 55.94) | 70.6% |  |  |  |  |  |  | 8.7 (6.7 – 12.5) | 94.3 (18.4 – 285.8) | 38 (26 – 48) |
|  |  | Non-severe (93) | 43.44 (40.4 – 46.5) | 68.8% |  |  |  |  |  |  | 5.9 (5 – 7.55) | 3.84 (0.71– 13) | 69 (44 – 99) |
