# Supplementary material for: Prevalence of severe Plasmodium knowlesi infection and risk factors related to severe complications compared with non-severe P. knowlesi and severe P. falciparum malaria: a systematic review and meta-analysis
Source: Infect Dis Poverty. 2020 Jul 29;9:106. doi: 10.1186/s40249-020-00727-x (PMC7392650; doi:10.1186/s40249-020-00727-x)
Supplement: Supplementary file 4 — Additional file 4: Table S3. Characteristic of severe Plasmodium knowlesi and P. falciparum. [file 40249_2020_727_MOESM4_ESM.docx]

**Table S3** Characteristic of severe *P.knowlesi* and *P.falciparum*

| No. | Author |  | Age | Parasite count,  ×10^3^/μL | Hemoglobin,  g/dL, mean (SD) | Leukocyte  count, ×10^3^/μL | Platelet count,  ×10^3^/μL | Creatinine, μmol/L | Sodium, mmol/L,  mean (SD) | Bilirubin, μmol/L | Glucose, mmol/L | Albumin, g/dL,  mean (SD) | AST, IU/L | ALT, IU/L |
| --- | --- | --- | --- | --- | --- | --- | --- | --- | --- | --- | --- | --- | --- | --- |
| 1. | Barber et al., 2013 | Severe Pk (38) | Median 55 (20–74) | 80.4 (25.9–  168.3) | 12.1 (1.82) | Total 6.60 (5.60–9.80)  Neutrophils 4.70 (3.49–7.57) | 29 (20–49) | 141 (101–213) | 131 (4.59) | 42.1 (26–66.8) | 7.7 (6.4–9.5) | 26.6 (5.32) | 57.5 (44–79) | 35.5 (19–51) |
|  |  | Severe Pf (13) | Median 33 (13–54) | 72.3 (27.9–  273.9) | 12.2 (3.09) | Total 5.0 (3.7–7.2)  Neutrophils 3.02 (2.29–4.52) | 24 (19–63) | 106 (80–184) | 130 (5.31) | 54.4 (27.1–76.7) | 6.2 (3.9–8.3) | 26.1 (4.39) | 46 (25–67) | 26 (18–60) |
| 2. | Barber et al., 2017 | Severe Pk (47) | 55 (20–81) | 104  (21.7–168.3) | NA | Neutrophils 4.3 (2.9–6.6) | 32 (20–57) | 145 (110–213) | NA | NA | NA | NA | 58 (39–103) | NA |
|  |  | Severe Pf (21) | 33 (13–60) | 44.3  (9.08–237.9) | NA | Neutrophils 3.1 (2.4–4.5) | 38 (22–63) | 125 (85–194) | NA | NA | NA | NA | 48 (28–72) | NA |
| 3. | Cox-Singh et al., 2011 | Severe Pk (9) | 61:12.2 (36 - 73) | 21.7 (4.3 – 148.3) | 11.8 (10.6 -13.3) | Total 7.4 (4.85- 11)  Neutrophils 3.7 (3.35 – 7.95) | 35(25- 54.5) | 185(107.6 - 325.5) | NA | 21.2(1.35- 55.32) | 6.3(6.2 - 7.0) | NA | 56(44.25 - 66) | NA |
|  |  | Severe Pf (5) | 33: 15.6 (15 - 49) | 73.9 (21.7- 242) | 10.7 (6.8 - 13.7) | Total 8.7(2.3- 11)  Neutrophils 4.35(1.40– 9) | 57 (42 – 164.5) | 82(44.5 - 109.5) | NA | 30.7(24.4 - 105.25) | 7.5 (6.1 - 8.3) | NA | 60(46.25 - 79.5) | NA |
| 4. | Daneshvar et al., 2009 | SeverePk (8) | 58.4 (36-73) | NA | NA | NA | NA | NA | NA | NA | NA | NA | NA | NA |
|  |  | Severe Pf | NA | NA | NA | NA | NA | NA | NA | NA | NA | NA | NA | NA |
| 5. | Grigg et al., 2018 | Severe Pk (28) | Median 53 (43–64) | 42.2 (17.2–103.6 | NA | NA | NA | NA | NA | NA | NA | NA | NA | NA |
|  |  | Severe Pf (5) | Median 14 (2-16) | 297 (85.5–635.4) | NA | NA | NA | NA | NA | NA | NA | NA | NA | NA |
| 6. | William et al., 2011 | Severe Pk | 57 (22–84) | NA | NA | NA | NA | NA | NA | NA | NA | NA | NA | NA |
|  |  | Severe Pf | NA | NA | NA | NA | NA | NA | NA | NA | NA | NA | NA | NA |
| 7. | Willmann et al., 2012 | Severe Pk | 49.59 (43.24 – 55.94) | NA | NA | NA | NA | NA | NA | NA | NA | NA | NA | NA |
|  |  | Severe Pf | NA | NA | NA | NA | NA | NA | NA | NA | NA | NA | NA | NA |
