# Supplementary figures and images for: Prevalence of severe Plasmodium knowlesi infection and risk factors related to severe complications compared with non-severe P. knowlesi and severe P. falciparum malaria: a systematic review and meta-analysis
Source: Infect Dis Poverty. 2020 Jul 29;9:106. doi: 10.1186/s40249-020-00727-x (PMC7392650; doi:10.1186/s40249-020-00727-x)

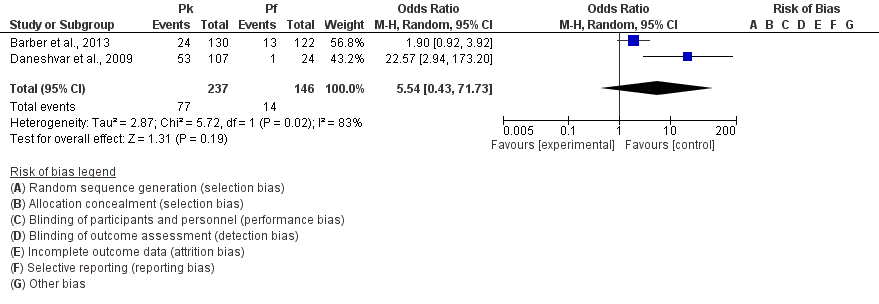

Supplement: Supplementary file 5 — Additional file 5: Figure S1. The proportion of farmers infected with Plasmodium knowlesi and P. falciparum. [file 40249_2020_727_MOESM5_ESM.png]

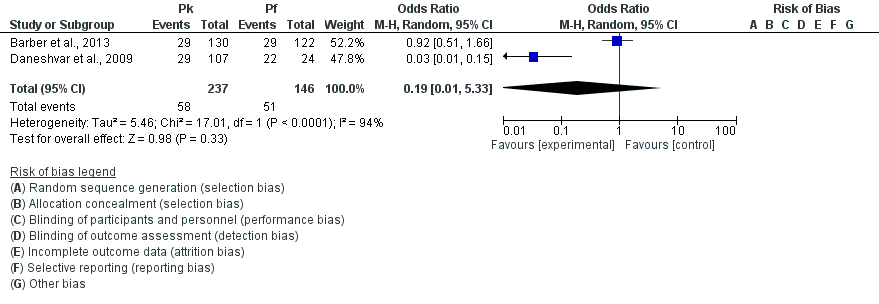

Supplement: Supplementary file 6 — Additional file 6: Figure S2. The proportion of plantation workers infected with Plasmodium knowlesi and P. falciparum. [file 40249_2020_727_MOESM6_ESM.png]
